# Medication adherence and its determinants among Brucellosis patients in China: A multi-center follow-up study

**DOI:** 10.1371/journal.pntd.0014741

**Published:** 2026-10-05

**Authors:** Qiaoshan Lu, Huijie Qin, Shijian Zhou, Sihan Li, Junyuan Chen, Qiulan Chen, Xianyan Tang

**Affiliations:** 1 Department of Epidemiology and Biostatistics, School of Public Health, Guangxi Medical University, Nanning, China; 2 National Key Laboratory of Intelligent Tracking and Forecasting for Infectious Diseases, Chinese Center for Disease Control and Prevention (Chinese Academy of Preventive Medicine), Beijing, China; 3 The First Affiliated Hospital of Guangxi Medical University, Nanning, China; Colorado State University, UNITED STATES OF AMERICA

## Abstract

**Background:**

Brucellosis patients in China face challenges in adhering to prescribed oral medication regimens during home-based treatment. Improving adherence is critical for disease control and relapse prevention. However, systematic studies on medication adherence and its determinants among Chinese brucellosis patients remain scarce. This study aimed to assess medication adherence and identify its multilevel determinants in this population.

**Methods:**

A multi-center study was conducted across six prefectures in China from December 2019 to August 2021. Demographic and clinical data were collected via questionnaires and medical records. Medication adherence was evaluated using the Chinese Morisky-8 Medication Adherence Scale (C-MMAS-8) at 6 and 12 weeks of treatment. Multilevel logistic regression models were employed to identify determinants of adherence.

**Results:**

Among 820 enrolled patients, 801 (49.49 ± 11.51 years) and 426 (50.81 ± 11.11 years) completed follow-ups at 6 and 12 weeks, respectively. Adherence scores declined significantly from 8 (IQRs: 5.75–8), (53.18% optimal adherent) at 6 weeks to 7 (IQRs: 5.5–8), (41.31% optimal adherent) at 12 weeks (*P* < 0.01). Quality of life scores correlated positively with adherence scores at both follow-ups (r=0.28 and 0.21; *P* < 0.01). Compared with inner Mongolia, medication adherence in Xinjiang (OR=2.14, 95% CI: 1.01–4.51, *P* < 0.05) and Shandong (OR=2.94, 95% CI: 1.41–6.15, *P* < 0.01) was significantly high. Key determinants included social support (OR=1.06, 95% CI: 1.01–1.11), prior brucellosis episodes (OR=0.42, 95% CI: 0.18–0.99), and concomitant use of hepatoprotective medications (OR=0.39, 95% CI: 0.23–0.67).

**Conclusions:**

Nearly half of brucellosis patients exhibited sub-optimal medication adherence, with adherence declining over time and significantly regional difference in optimal adherence. Enhancing social support, prioritizing patients with prior infection history for tailored education, and ensuring safe and rational medication use represent key strategies to improve adherence. Tailored interventions incorporating these elements are urgently needed to optimize disease management and reduce the risk of chronicity.

## Author summary

Brucellosis requires prolonged oral antibiotics, yet home-treated patients often struggle with adherence, increasing relapse and chronicity risk. We enrolled 820 patients across six Chinese prefectures, and 801 of these patients completed the 6-week follow-up. Of the 801 participants, 346 completed the standard 6-week regimen, while 455 continued to the 12-week stage. In conclusion, 426 of the 12-week regimen completed the 12-week follow-up. We found that optimal adherence dropped from 53.2% at week 6 to 41.3% at week 12, and correlated positively with quality of life at both time points. Compared with Inner Mongolia, adherence was significantly higher in Xinjiang (OR=2.14, 95% CI: 1.01–4.51, *P* < 0.05) and Shandong (OR=2.94, 95% CI: 1.41–6.15, *P* < 0.01). Multilevel regression identified three determinants: social support (OR=1.06) promoted adherence, while prior episodes (OR=0.42) and hepatoprotective use (OR=0.39) were linked to poorer adherence, possibly due to fatigue or pill burden. Nearly half failed to maintain adherence over time; inter-regional variation calls for context-sensitive strategies. Strengthening social support, targeted education for recurrent cases, and rationalizing medication use are essential to improve completion and reduce chronic brucellosis burden in China.

## Introduction

Brucellosis, a globally significant zoonotic disease, severely undermines livestock productivity and human health, particularly in resource-limited settings [1]. An estimated 3.5 billion individuals worldwide are at risk of infection, with developing countries bearing the highest burden [2]. In China, human brucellosis incidence has surged in recent years, disproportionately affecting populations from low- and middle-income socioeconomic strata [3,4]. The disease exacerbates cycles of poverty, as high out-of-pocket medical expenses impose crippling financial burdens on households and healthcare systems [5].

Brucellosis has the potential to infiltrate multiple organ systems, presenting with a plethora of clinical manifestations, such as hepatosplenomegaly, osteoarticular involvement, and neuro-brucellosis. *Brucella* is an intracellular parasite capable of evading host immune responses through multiple mechanisms, the disease often requires prolonged antibiotic regimens [4], yet premature discontinuation due to poor adherence remains a major barrier to effective treatment [6]. In China, the rifampicin–doxycycline regimen, as recommended by the World Health Organization (WHO) for a duration of 6–12 weeks, constitutes the standard treatment for uncomplicated acute brucellosis. However, patients with chronic infections and/or complications may need a 12–48 week treatment course, plus one or more antibiotics other than the primary regimen (i.e., combination of rifampicin and doxycycline) [7]. In the context of China’s healthcare system, the shortening of hospitalization duration has necessitated that patients continue oral medication at home, thereby precipitating a series of challenges, including complex medication regimens, adverse drug reactions, and limited follow-up support. All these challenges jeopardize medication adherence. Non-adherence drives disease progression, complications, and elevated healthcare costs. Crucially, patients completing full treatment cycles exhibit significantly lower risks of chronicity compared to those discontinuing therapy prematurely [8]. Chronic brucellosis profoundly impairs work capacity and quality of life, perpetuating economic strain on individuals, families, and society [5]. These realities underscore the urgent need to investigate medication adherence patterns and their determinants, enabling tailored interventions to optimize therapeutic outcomes and mitigate public health impacts.

While the WHO emphasizes adherence as a cornerstone of therapeutic success [9], few studies have systematically evaluated adherence patterns or determinants in Chinese brucellosis patients, limited to small-scale, single-center studies with questionable generalizability [8]. To address this gap, our multi-center study employed a large sample and follow-up investigation across diverse clinical settings in China. By systematically evaluating adherence behaviors and multilevel influencing factors, this work aims to provide a scientific basis and data support for optimizing the medication management strategy and formulating an optimal follow-up policy for brucellosis cases in China.

## Methods

### Ethics approval and consent to participate

The study protocol received approval from the Research Ethics Review Committee of the Chinese Center for Disease Control and Prevention (Approval number: 201942). Prior to the investigation, all participants provided signed informed consent. For child participants, formal informed written consent was obtained from their parents or legal guardians.

### Study design and setting

A multi-center study was conducted in six cities in China, namely Chifeng and Bayannur prefecture-level city in Inner Mongolia Autonomous Region, Ili and Changji prefecture-level city in Xinjiang Uygur Autonomous Region, and Jinan and Dongying prefecture-level city in Shandong Province, from December 2019 to August 2021. These prefectures were selected based on their high incidence rates of brucellosis, being among the top 10% in their respective provincial-level administrative divisions (PLADs). All project sites were designated medical institutions for brucellosis treatment and had the necessary capacity to conduct the required examinations and laboratory tests. The study protocol was approved by the Research Ethics Review Committee of the Chinese Center for Disease Control and Prevention (Approval number: 201942), and signed informed consent was obtained from all participants prior to the investigation.

### Sample size

Sample size estimation was based on the formula for confidence interval estimation of a single population proportion. A review of the literature revealed that the adherence rate of patients with brucellosis has rarely been reported in the last decade. Therefore, we chose a conservative estimate of 50% to ensure the maximum sample size. Considering confidence levels: 95% confidence intervals (CIs) formula was Exact (Clopper-pearson), the width of the CIs (two-sided) was 10%, the dropout rate was 5%, the design effect was 2, the minimum sample size was 809 individuals. Ultimately, a total of 820 brucellosis patients were included in the study.


$$\begin{array}{c} n=\frac{Z_{\text{1}-\frac{\alpha }{\text{2}}}^{\text{2}}\times p(\text{1}-p)}{\sigma^{\text{2}}}\times deff\times \frac{\text{1}}{\text{1}-\text{dropout rate }} \end{array}$$

### Sampling and participants

A multi-stage random sampling method was utilized to select specific hospitals and patients with human brucellosis. In regions of Inner Mongolia, Xinjiang, and Shandong with high incidence rates, six county-level designated medical institutions were chosen. From December 2019 to August 2021, doctors from these designated medical institutions enrolled eligible brucellosis patients who met the criteria, following the principle of informed consent. Enrollment continued until the desired sample size was achieved.

The inclusion criteria for cases were as follows: (1) patients diagnosed with brucellosis; (2) aged 15 years and above; and (3) provided informed consent and willingly participated in the study.

The criteria for case exclusion were: (1) infected with the human immunodeficiency virus (HIV), undergoing chemotherapy, having other immune system disorders, or suffering from other severe illnesses; (2) suffering from mental disorders, deafness and other disorders resulting in poor communication and inability to cooperate with the investigation; (3) patients whose address is unknown, who cannot be contacted, who cannot cooperate with the investigation, and who have died; and (4) pregnant woman.

The definition of confirmed cases of brucellosis includes patients who have both a history of exposure and clinical symptoms, and meet one of the following criteria: (1) Isolation and culture of *Brucella* bacteria from the patient’s blood or other specimens; (2) A serum agglutination test (SAT) titer of ≥1:100 (++), or a duration of illness of over one year with a titer of ≥1:50 (++); and (3) A Coombs test titer of ≥1:400 (++).

### Data collection and definitions

Following informed consent acquisition, trained physicians administered standardized questionnaires to brucellosis-diagnosed patients through face-to-face interviews. The instrument systematically captured demographic characteristics (gender, age, ethnicity, occupation, educational attainment), social support metrics, and clinical parameters extracted from the Hospital Information System (HIS), including: pre-existing comorbidities, brucellosis history, clinical manifestations (e.g., fever, arthralgia, fatigue), complications, therapeutic approaches (outpatient/inpatient care), and pharmacological regimens. A dual-phase quality control protocol was implemented: all interviewers completed competency-based training on questionnaire administration and clinical data abstraction; and then supervisory staff conducted systematic reviews of completed questionnaires, while data management specialists performed completeness checks and logic validation.

Medication adherence was quantified using the validated *Chinese Morisky-8 Medication Adherence Scale* (C-MMAS-8) [10]. This 8-item Likert-type instrument demonstrates established reliability (Cronbach’s α = 0.83) and criterion validity across chronic disease populations. Using standard thresholds, adherence was classified as high (score = 8), moderate (score 6–7.99), or low (score < 6). For chronic diseases, the maintenance of steady-state plasma concentrations is of paramount importance; even one missed dose can cause significant trough-level fluctuations that undermine clinical effectiveness. In view of the unique treatment paradigm of brucellosis, adherence was dichotomous as optimal (score = 8) vs. suboptimal (score <8).

Social support was assessed via the *Social Support Rating Scale* (SSRS; Xiao Shuiyuan, 1986), a 10-item multidimensional tool measuring objective support, subjective support, and support utilization. Total scores (range 12–66) were analyzed as continuous variables, with higher values indicating stronger support networks.

Health-related quality of life was evaluated using the *European Quality of Life Visual Analogue Self-Assessment Scale* (EQ-VAS) component of the EuroQol-5D instrument. Participants self-rated current health status on a 100mm vertical visual analog scale (0 = worst imaginable health state; 100 = best imaginable), with quantitative scores recorded at 6- and 12-week follow-ups via in-person or telemedicine assessments.

Hepatoprotective therapy encompassed pharmacologic agents with documented liver-protective properties, specifically: Hepatoprotective tablets (compound formulation: inositol, choline, methionine); Silymarin capsules (standardized to ≥80% silibinin); and Compound glycyrrhizin tablets (containing glycyrrhizin, cysteine, and glycine)

### Statistical analysis

Data storage was done using EpiData version 3.1 (EpiData Association, Odense, Denmark). The normality assumption for quantitative variables was assessed using the Kolmogorov-Smirnov (KS) test with Lilliefors correction for significance. Non-normally distributed variables were described using median and inter-quartile ranges (IQRs). Categorical variables were described using frequency and percentage. Differences in proportions were tested using Pearson’s x^2^ test or Fisher’s exact test. Correlation analyses were conducted using either Pearson’s or Spearman’s correlation coefficient.

As the research was conducted in six hospitals and measure the outcome of medication adherence for two times, i.e., at 6-week and 12 weeks after the initiation of therapy, the data had three hierarchical structure: follow-up time level; patient-time level and hospital-patient-time level. And therefore, the multilevel logistic regression model was employed to identify the determinants of medication adherence in brucellosis patients using the “R2MLwiN” package in R software version 3.4.2 (R Project for Statistical Computing, Vienna, Austria). Univariate multilevel logistic regression was used to screen variables, considering *P* < 0.2 as statistically significant. Factors selected from the screening were included in a multivariate multilevel analysis. Multiple categorical variables were included in the model as dummy variables. Definition and assignment of dependent and independent variables see S1 Table.

The data in this study constitute an unbalanced panel dataset, with sample sizes differing across follow-up visits. Model estimation was performed using available-case analysis, incorporating observed data at each time point without imputation of missing values. For the patients who had completed the standard 6-week regimen and subsequently discontinued treatment per protocol, medication exposure had ceased by week 12. Consequently, their adherence outcomes at that visit were not defined and were treated as structurally missing rather than missing at random. These outcomes were thus coded as ‘Not Applicable’ (NA).

For best fit, the variances of the null model were compared at different levels (please see the S2 Table. Parameter estimation for null models at different levels). In the one-level model (i.e., time level), constant variance was significant, implying that the multilevel model should be fitted for the hierarchical structure of the data. In the two-level model (i.e., patient-time level), variance at the patient level was significant, indicating that the random effect of patient-level factors existed, and nearly 53% of the time-level variance was attributed to that at the patient level. The variance partition coefficient was 53%. In the three-level model (i.e., hospital-patient-time level), hospital-level variance was not significant (*P* > 0.05). Thus, two-level logistic regression models were developed to fit the data in this study.

## Results

We enrolled a total of 820 participants diagnosed with human brucellosis. The study period spanned from December 2019 to August 2021. Among the participants, 329 (40.12%) were from Xinjiang, 241 (29.39%) from Shandong, and 250 (30.49%) from Inner Mongolia. Of these, 801 (97.7%) completed the 6-week follow-up: 346 had completed their standard 6-week regimen and were not required to continue, while the remaining 455 were prescribed an extended 12-week course. Among the 455, 426 (93.6%) successfully completed the 12-week follow-up. Please refer to Fig 1 and S3 Table for details regarding the specific follow-up process and distribution of patient items.

**Fig 1:**
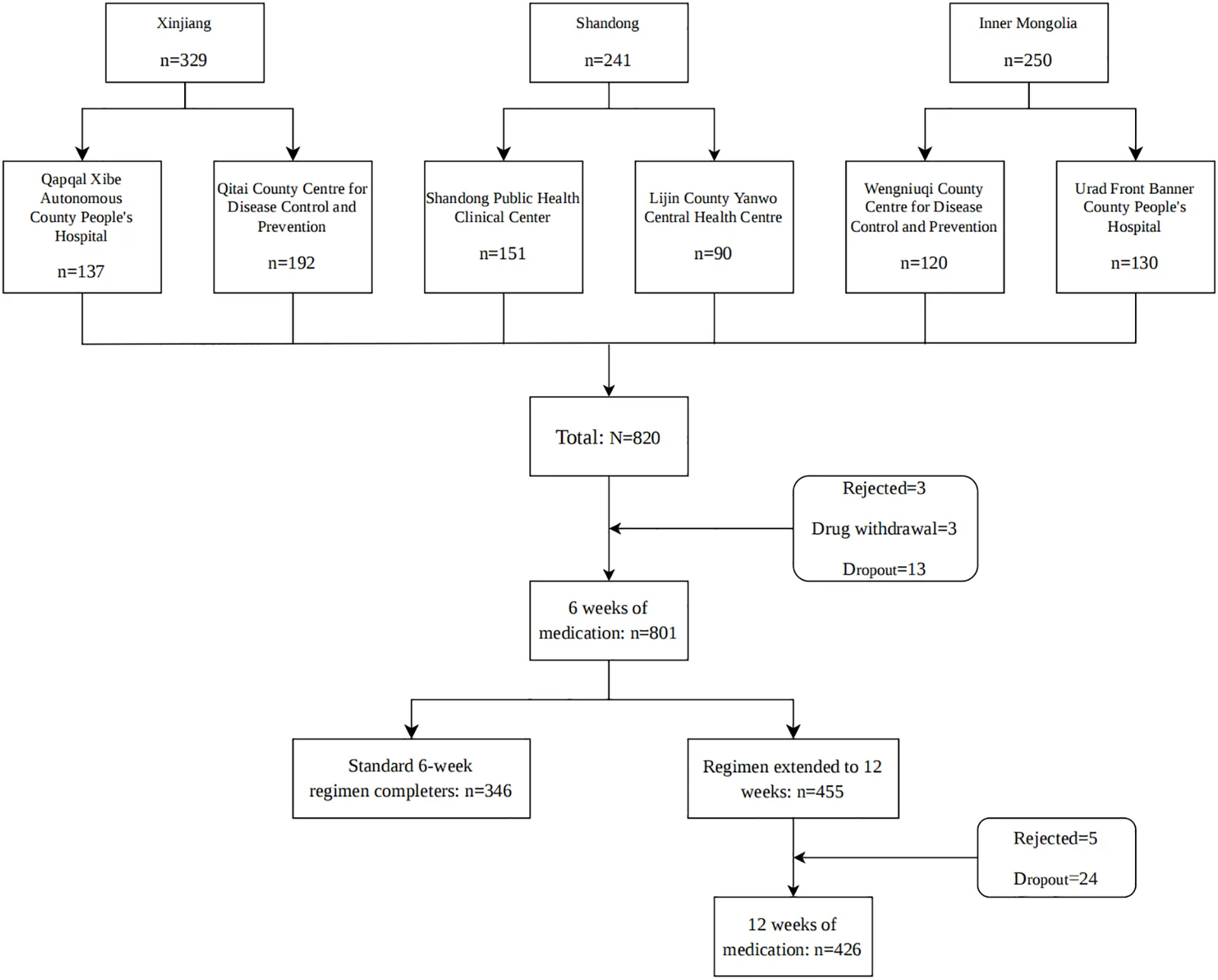
Patient inclusion and follow-up process.

### Demographic and epidemiological characteristics

Demographic and epidemiological characteristics, stratified by follow-up time, are displayed in Table 1. The average age of the patients at the initial follow-up was 49.49 ± 11.51 years. A total of 572 (71.41%) patients were male, 214 (26.72%) were minority ethnicity, 706 (88.14%) were farmers or herders, 350 (43.70%) had received no more than primary-level education, and the average score on the social support scale was 43.80 ± 5.79. The average age of the patients in the second follow-up was 50.81 ± 11.11 years. A total of 311 (73.00%) patients were male, 83 (19.84%) were ethnic minorities, 372 (87.32%) were farmers or herders, 180 (42.25%) had received no more than primary-level education, and the average score on the social support scale was 44.12 ± 5.83. The two follow-up groups differed significantly only in terms of ethnicity and medication regimen (*P* < 0.01 and *P* = 0.02), without significant differences observed in other demographic or clinical characteristics (*P* > 0.05).

**Table 1:** Demographic characteristics of brucellosis patients n (%).

Variables	6 weeks	12 weeks	$x^{2}$ */t*	*P* value
Region			24.85	**<0.01**
Xinjiang	323 (40.32)	161 (37.79)		
Shandong	238 (29.71)	180 (42.25)		
Inner Mongolia	242 (30.21)	85 (19.95)		
Age (years)	49.49 ± 11.51	50.81 ± 11.11	-1.92	0.06
Gender				
Male	572 (71.41)	311 (73.00)	0.35	0.55
Female	229 (28.59)	115 (27.00)		
Ethnicity				
Han ethnicity	587 (73.28)	343 (80.52)	7.93	**0.01**
Minority ethnicity	214 (26.72)	83 (19.84)		
Occupation				
Farmer& Herdman	706 (88.14)	372 (87.32)	0.19	0.91
Animal and animal products processing management	43 (5.37)	25 (5.87)		
Other occupations^a^	52 (6.49)	29 (6.81)		
Education				
Primary school or below	350 (43.70)	180 (42.25)	0.28	0.87
Junior high school	355 (44.32)	192 (45.07)		
High school or above	96 (11.99)	54 (12.68)		
Social support score	43.80 ± 5.79	44.12 ± 5.83	-0.92	0.36
Family history of brucellosis				
Yes	184 (22.97)	87 (32.10)	1.05	0.31
No	617 (77.03)	339 (35.46)		
Underlying disease				
Yes	259 (32.33)	130 (30.52)	0.43	0.52
No	542 (67.67)	296 (69.48)		
Prior brucellosis episodes				
Yes	87 (10.86)	45 (10.57)	0.03	0.87
No	714 (89.14)	381 (89.44)		
Complications				
Yes	361 (45.07)	211 (49.53)	2.23	0.14
No	440 (54.93)	215 (50.47)		
Fever				
Yes	438 (54.68)	244 (57.28)	0.76	0.38
No	363 (45.32)	182 (42.72)		
Arthralgia				
Yes	673 (84.02)	347 (81.46)	1.30	0.25
No	128 (15.98)	79 (18.54)		
Fatigue				
Yes	576 (71.91)	291 (68.31)	1.74	0.19
No	225 (28.09)	135 (31.69)		
Hospitalization				
Yes	573 (71.54)	322 (75.59)	2.31	0.13
No	228 (28.46)	104 (24.41)		
Prescription				
Rifampicin + Doxycycline	319 (39.83)	134 (31.46)	8.37	**0.02**
Rifampicin + Doxycycline + other antibiotics	405 (50.56)	246 (57.75)		
Monotherapy or non-antibiotic drug regimen	77 (9.61)	46 (10.80)		
Hepatoprotective medications				
Yes	483 (60.30)	260 (61.03)	0.06	0.80
No	318 (39.70)	166 (38.97)		
Comorbidities and concomitant medications				
Yes	87 (10.86)	53 (12.44)	0.69	0.41
No	714 (89.14)	373 (87.56)		

^a^Other occupations include staff, student, teacher, and other non-occupational population with brucellosis

### Clinical characteristics

At baseline, among the 801 enrolled brucellosis patients, 87 individuals (10.86%) reported a prior brucellosis episodes, while 259 patients (32.33%) presented with comorbidities. The most common clinical manifestations were arthralgia (n = 673, 84.02%), fatigue (n = 576, 71.91%), and fever (n = 438, 54.68%). Complications were observed in 361 patients (45.07%). Treatment regimens included rifampicin-doxycycline combination therapy in 319 patients (39.83%), rifampicin-doxycycline with additional antibiotics in 405 patients (50.56%), and non-antibiotic therapies in 77 patients (9.61%). Hepatoprotective medications were administered to 483 patients (60.30%), while 87 patients (10.86%) received medications for comorbid conditions (Table 1).

### Social support

At baseline, all patients underwent an assessment of social support. Among the 801 patients available at the first follow-up, the baseline social support score was 43.80 ± 5.79, with objective support scoring 9.23 ± 2.38, subjective support scoring 26.51 ± 3.22, and support utilization scoring 8.06 ± 2.28. For the 426 patients available for the second follow-up, the baseline social support score was 44.12 ± 5.83, with objective support scoring 9.34 ± 2.26, subjective support scoring 26.49 ± 3.14, and support utilization scoring 8.30 ± 2.29 (please see the S4 Table).

### Medication adherence in brucellosis patients

After 6 weeks of medication, the medication adherence score of brucellosis patients was 8 (IQRs: 5.75–8) and the proportion of optimal adherence was 53.18%. After 12 weeks of medication, the medication adherence score of brucellosis patients was 7 (IQRs: 5.5–8) and the proportion of optimal adherence was 41.31%. There were significant differences in both the adherence scores and the proportions of adherence between the two time points (all *P* < 0.01; Fig 2).

**Fig 2:**
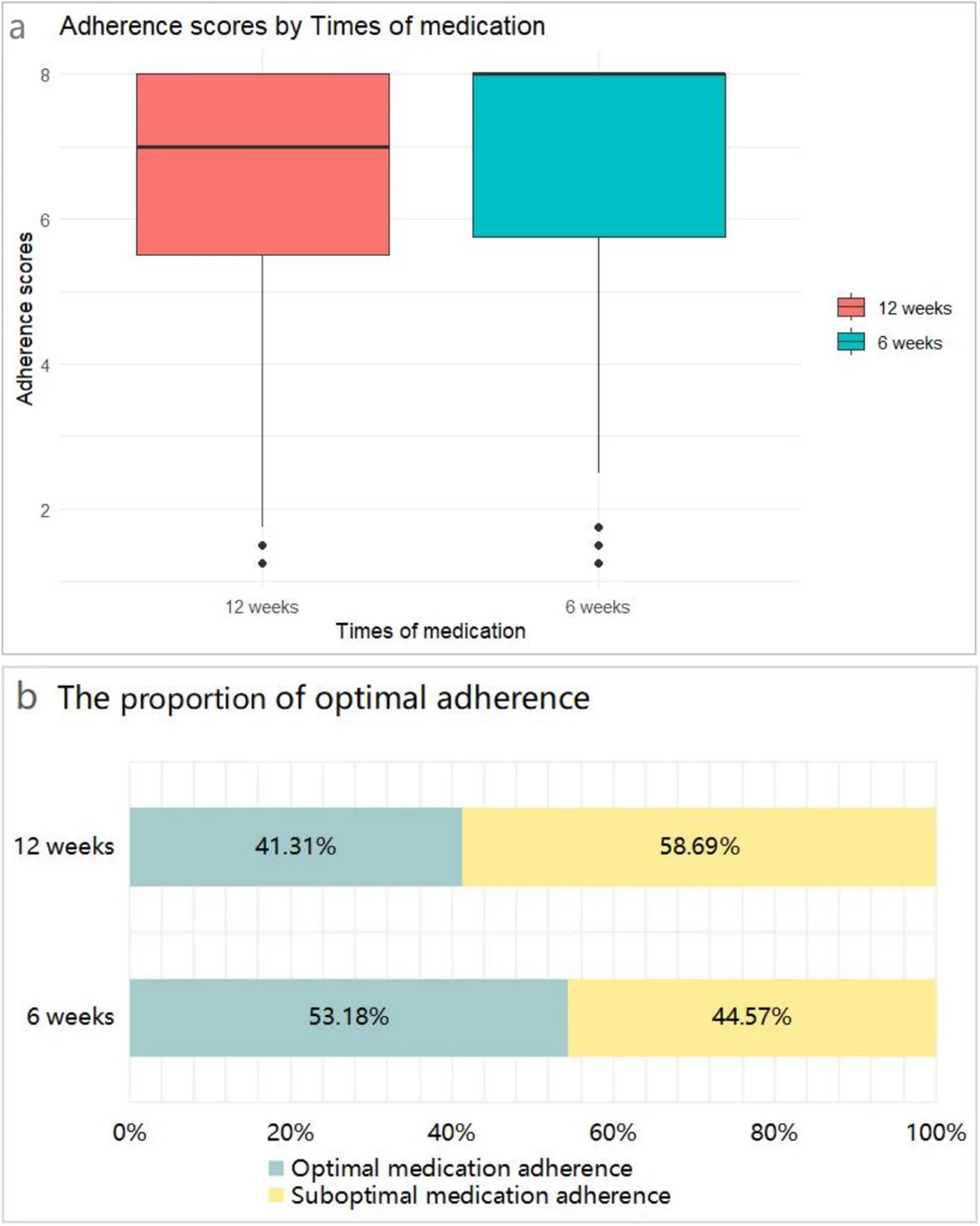
Medication adherence in brucellosis patients.

### Determinants of medication adherence in brucellosis patients (Table 2)

The univariate multilevel logistic regression analysis revealed that several factors were significantly associated with medication adherence among brucellosis patients, including region (*P* < 0.01), ethnicity (*P* = 0.01), social support score (*P* < 0.01), family history of brucellosis (*P* = 0.03), arthralgia (*P* = 0.05), prior brucellosis episodes (*P* < 0.01), monotherapy or non-antibiotic regimen (*P* < 0.01), and concomitant use of hepatoprotective medications (*P* < 0.01).

**Table 2:** Univariate and multivariate multilevel logistic regression analysis of medication adherence in brucellosis patients.

Variables	Univariate analysis		Multivariate analysis	
	OR (95% CI)	*P* value*	OR (95% CI)	*P* value*
Region				
Inner Mongolia	1.00			
Xinjiang	3.22(1.21-8.60)	**<0.01**	2.14(1.01-4.51)	0.05
Shandong	4.62(1.73-12.33)	**<0.01**	2.94(1.41-6.15)	**<0.01**
Age (years)	–	0.11	0.99 (0.97-1.01)	0.41
Gender				
Male	1.00			
Female	0.99 (0.63-1.55)	0.95	–	–
Education				
Primary school or below	1.00			
Junior high school	1.04 (0.68-1.59)	0.87	–	–
High school or above	1.53 (0.79-2.95)	0.21	–	–
Ethnicity				
Han ethnicity	1.00			
Minority ethnicity	1.83 (1.13-2.95)	**0.01**	1.16 (0.60-2.25)	0.66
Occupation				
Farmer& Herdman	1.00			
Animal and animal products processing management	1.08 (0.44-2.66)	0.87	0.50(0.17-1.52)	0.22
Other occupations^a^	2.22 (0.95-5.20)	0.07	1.79 (0.65-4.92)	0.26
Social support score^b^	1.07 (1.03-1.10)	**<0.01**	1.06 (1.01-1.11)	**<0.01**
Family history of brucellosis				
No	1.00			
Yes	0.58 (0.35-0.94)	**0.03**	0.58 (0.32-1.06)	0.08
Arthralgia				
No	1.00			
Yes	1.76 (1.01-3.06)	0.05	0.63 (0.32-1.23)	0.17
Fever				
No	1.00			
Yes	0.68 (0.45-1.02)	0.06	0.65 (0.39-1.07)	0.09
Fatigue				
No	1.00			
Yes	1.27 (0.82-1.98)	0.29	–	–
Complications				
No	1.00			
Yes	0.76 (0.51-1.13)	0.18	0.61 (0.36-1.03)	0.06
Prior brucellosis episodes				
No	1.00			
Yes	0.29 (0.14-0.58)	**<0.01**	0.42 (0.18-0.99)	**<0.05** ^c^
Hospitalization				
No	1.00			
Yes	0.66 (0.42-1.03)	0.07	1.01(0.55-1.85)	0.98
Underlying disease				
No	1.00			
Yes	1.17 (0.76-1.80)	0.47	–	–
Prescription				
Rifampicin + Doxycycline	1.00			
Rifampicin + Doxycycline + other antibiotics	0.67 (0.44-1.04)	0.07	0.62 (0.32-1.20)	0.16
Monotherapy or non-antibiotic regimen	0.22 (0.10-0.47)	**<0.01**	0.40 (0.14-1.15)	0.09
Hepatoprotective medications				
No	1.00			
Yes	0.32 (0.21-0.48)	**<0.01**	0.39 (0.23-0.67)	**<0.01**
Comorbidities and concomitant medications				
No	1.00			
Yes	1.04 (0.55-1.97)	0.89	–	–
**Variance components**				
Patient level	–	–	127.74	<0.01
Time level	–	–	1.00	1.00

^a^Other occupations include staff, student, teacher, and other non-occupational population with brucellosis

^b^The reported OR is per 1-point increase on the SSRS scale

^c^*P*=0.0476

OR, odds ratio. * Penalized Quasi-likelihood (PQL) test.

We conducted multivariate multilevel logistic regression analyses to identify independent factors associated with adherence. With respect to regional difference, compared with Inner Mongolia, the odds ratios (ORs) for Xinjiang and Shandong was 2.14 (95% CI: 1.01–4.51, *P* = 0.05) and 2.94 (95% CI: 1.41–6.15, *P* < 0.01), respectively. Our findings also indicate that several factors were independently associated with medication adherence among brucellosis patients, including social support score (OR=1.06, 95% CI: 1.01–1.11, *P* < 0.01), prior brucellosis episodes (OR=0.42, 95% CI: 0.18–0.99, *P* < 0.05), and hepatoprotective medications (OR=0.39, 95% CI: 0.27–0.67, *P* < 0.01).

### Association of medication adherence with quality of life scores

The results of our study demonstrated that the median EQ-VAS scores were 85.00 (IQR: 80.00–90.00) at 6 weeks and 90.00 (IQR: 80.00–93.00) at 12 weeks after medication initiation. A positive correlation was observed between the medication adherence scores obtained during both follow-up assessments and the EQ-VAS scores, with correlation coefficients of 0.28 and 0.21, respectively, and both correlations were significant (*P* < 0.01; Table 3).

**Table 3:** Correlation analysis between medication adherence and EQ-VAS in brucellosis patients.

		Medication adherence at 6 weeks		Medication adherence at 12 weeks	
	M (P25, P75)	$r_{s}$	*P* value	$r_{s}$	*P* value
**EQ-VAS at 6 weeks**	85.00 (80.00, 90.00)	0.28	**<0.01**	–	
**EQ-VAS at 12 weeks**	90.00 (80.00, 93.00)	–		0.21	**<0.01**

## Discussion

This study systematically examined medication adherence and its multilevel determinants among Chinese brucellosis patients, aiming to inform targeted strategies for optimizing therapeutic management. Our findings revealed that nearly half of the patients exhibited sub-optimal adherence to prescribed regimens, with optimal adherence rates declining substantially from 53.18% at 6 weeks to 41.31% at 12 weeks (*P* < 0.01), and that significant regional difference in optimal adherence existed. Key determinants included social support, prior brucellosis episodes, and concomitant use of hepatoprotective medications. These results align with global trends in chronic infectious diseases but highlight unique challenges within China’s healthcare context.

The observed sub-optimal adherence rates (44.57% at 6 weeks; 58.69% at 12 weeks) among brucellosis patients in China, exceed global averages (22.3%) and previously reported Chinese data (44%) for taking antibiotics against acute infections [11]. As *Brucella* is an intracellular parasite with multiple mechanisms to evade host immune responses, a more prolonged therapeutic regimen combining specific antibiotics is required to achieve complete eradication of the pathogen, compared to those acute infectious diseases [4]. This differnce underscores systemic gaps in China’s post-discharge care infrastructure for brucellosis case management, including limited follow-up systems and insufficient patient education. Although China has established a directly observed treatment (DOT) system for tuberculosis patients through primary healthcare and public health institutions, the necessity of implementing a similar supervised medication system for brucellosis remains under-recognized, partly due to the absence of human-to-human transmission. Brucellosis patients, who exhibit rapid symptomatic relief (typically within 1–2 weeks) owing to high sensitivity to specific antibiotics, are generally treated at home or transition to home-based care after brief hospitalization. Home-based oral therapy, while cost-effective, often fails to address competing socioeconomic pressures (e.g., occupational demands, financial constraints) or provide continuous clinical guidance, exacerbating non-adherence [12,13]. The decline in adherence over time may reflect patients’ misperceptions of cure following rapid symptom resolution (85.20% within one week for acute cases), leading to premature discontinuation despite the necessity of prolonged therapy [14,15]. Notably, the positive correlation between EQ-VAS scores and adherence (*r* = 0.28, 0.21; both *P* < 0.01) reinforces bidirectional links between treatment behaviors and quality of life, as documented in chronic disease populations [16,17].

There were significantly regional difference in optimal adherence. In Xinjiang, a primary-care-based infectious disease management model has been shown to effectively mitigate treatment interruptions precipitated by pastoral activities. This model employs grid general practitioner services and systematic home follow-ups with supervised medication, thereby ensuring the continuity of healthcare services amidst pastoral lifestyles. In Shandong, the dense distribution of healthcare facilities, higher educational attainment, and the widespread use of Mandarin have collectively lowered healthcare costs and educational barriers. This indicates that, compared with static regional attributes, policy implementation and adaptive resource allocation may play a more critical role in shaping therapeutic adherence.

Social Support emerged as a critical facilitator, consistent with WHO frameworks emphasizing socioeconomic factors in adherence [9]. Social support can provide patients with material, emotional, and medication-related information support, occupies an important position in several health behavior-related theories and therefore is a key component of socioeconomic-related factors [9,18]. Enhanced social networks likely bolster self-efficacy through material aid, emotional reassurance, and medication-related guidance, aligning with health behavior models like the Information-Motivation-Behavioral Skills (IMB) framework [18]. Conversely, prior brucellosis episodes are inversely associated with adherence, likely reflecting disillusionment with prior treatments and diminished trust in providers [19,20]. Additionally, patients with recurrent infections often lack core self-management skills (e.g., medication scheduling), which may perpetuate poor adherence. This deficiency can perpetuate suboptimal adherence patterns, a phenomenon that aligns with the IMB model’s proposition that inadequate behavioural skills act as a barrier to adherence [21].

Standard treatment regimens for brucellosis mainly consist of doxycycline combined with rifampicin or streptomycin, all of which carry some degree of hepatotoxicity [22]. In clinical practice, patients who require hepatoprotective agents often already have drug induced liver injury (DILI). Systemic symptoms attributable to liver injury, such as nausea, anorexia, and fatigue, may be the primary drivers of dose reduction or treatment discontinuation in these patients [22]. Furthermore, hepatoprotective drugs, though mitigating rifampicin toxicity, increase financial strain and the number of medications taken (i.e., pill burden), fostering anxiety and treatment discontinuation [5,15,23]. These findings underscore the need for safe, simplified regimens tailored to socioeconomic realities.

Contrary to prior studies [24,25], socio-demographic variables (age, gender, education) were not significantly associated with adherence. This discrepancy may reflect our cohort’s homogeneity (predominantly rural, low-income farmers/herders) or measurement differences. Although previous studies have identified complications as adherence correlates [26], patients with complications in our study showed only a trend toward poorer adherence (OR=0.61, 95% CI: 0.36–1.03, *P* = 0.06), which did not reach significance partly due to the sample size.

## Limitations

This study has several limitations. First, the lack of adjustment for known confounders (e.g., treatment costs, insurance coverage) may have biased results [23]. Second, reliance on self-reported adherence (C-MMAS-8) risks recall and social desirability biases [27]. Third, the 12-week endpoint precluded assessment of long-term adherence dynamics. Fourth, the present study did not examine the causal relationship between quality of life and medication adherence. Further studies are warranted to establish the temporal sequence and causal direction of this association, and to determine whether baseline quality of life serves as an independent predictor of subsequent adherence behavior after adjusting for potential confounders. In addition, future research should employ mixed-methods designs, integrate economic indicators, and explore causal pathways using advanced modeling (e.g., Bayesian networks) [28].

### Policy implications

To effectively address these challenges and improve treatment compliance, we propose a series of targeted interventions. First and foremost, a strengthened follow-up system is crucial. Telehealth platforms should be implemented to enable remote monitoring of patients and provide educational resources. By leveraging modern technology, healthcare providers can closely track patients’ treatment progress, offer timely advice, and answer their questions [29]. High-risk groups, such as those with a prior history of brucellosis or a history of non-adherence, should be prioritized in this follow-up system. This approach can help identify potential issues early and ensure that patients stay on track with their treatment regimens.

Second, regimen optimization is essential. Development of new regimen to achieve shorter therapy period with less side effect is needed in the near future. Additionally, considering the financial burden of hepatoprotective agents on patients, subsidizing these drugs can alleviate financial barriers. This not only reduces the economic stress on patients but also encourages them to continue with their treatment, which is vital for achieving better treatment outcomes.

Lastly, community engagement plays a pivotal role. Establishing peer support networks can enhance the social capital of patients. In these networks, patients can share their experiences, offer mutual support, and learn from one another. This sense of community can combat the stigma often associated with brucellosis, which may otherwise prevent patients from seeking proper treatment or adhering to their medication regimens. By working together at the community level, we can create a more supportive environment for brucellosis patients, ultimately improving their treatment adherence and overall well-being.

## Conclusions

This study highlights alarmingly low rates of optimal adherence among Chinese brucellosis patients, driven by intersecting clinical, socioeconomic, and systemic factors. Multilevel interventions are required with some urgency in order to curtail the chronic nature of the disease and improve quality of life. These interventions should include providing safe and simplified regimens, leveraging social support, and integrating telehealth.

## Supporting information

S1 Table Definition and assignment of dependent and independent variab. (DOCX)

S2 Table Parameter estimation for null models at different levels. (DOCX)

S3 Table Distribution of study subjects n(%). (DOCX)

S4 Table Social support scores across different dimensions. (DOCX)

S5 Table The 8-item Morisky Medication Adherence Scale (MMAS-8). (DOCX)
