# Supplementary material for: Medication adherence and its determinants among Brucellosis patients in China: A multi-center follow-up study
Source: PLoS Negl Trop Dis. 2026 Oct 5;20(10):e0014741. doi: 10.1371/journal.pntd.0014741 (PMC13637894; doi:10.1371/journal.pntd.0014741)
Supplement: Supplementary file 1 [file pntd.0014741.s001.docx]

| **S1** **Table.** **Definition and assignment of dependent and independent variables** | |
| --- | --- |
| **Variables** | **Assignment** |
| Follow-up time level (Level 1)  At the 6-week mark of medication  At the 12-week mark of medication  Patient level (Level 2) |  |
| Age (years) | $\bar{x}\pm s$ |
| Gender | Male=0，Female=1 |
| Ethnicity | Han ethnicity=0，Minority ethnicity=1 |
| Occupation | Farmer& Herdman=1，Animal and animal products processing management=2，Other occupations^a^=3 |
| Education | Primary or below=1，Junior high school=2，High school or above=3 |
| Social support score | $\bar{x}\pm s$ |
| Family history of brucellosis | No=0，Yes=1 |
| Underlying disease | No=0，Yes=1 |
| Prior brucellosis episodes | No=0，Yes=1 |
| Complications | No=0，Yes=1 |
| Fatigue | No=0，Yes=1 |
| Fever | No=0，Yes=1 |
| Arthralgia | No=0，Yes=1 |
| Hospital level (Level 3) |  |
| Hospitalization | No=0，Yes=1 |
| Prescription | Rifampicin + Doxycycline=1，Rifampicin + Doxycycline + other antibiotics=2，Monotherapy or non-antibiotic regimen^*^=3 |
| Hepatoprotective medications | No=0，Yes=1 |
| Comorbidities and concomitant medications | No=0，Yes=1 |
| **Dependent variable** | Suboptimal adherence=0，Optimal adherent=1 |

^a^ Other occupations include staff, student, teacher, and other non-occupational population with brucellosis.
