# Supplementary material for: Medication adherence and its determinants among Brucellosis patients in China: A multi-center follow-up study
Source: PLoS Negl Trop Dis. 2026 Oct 5;20(10):e0014741. doi: 10.1371/journal.pntd.0014741 (PMC13637894; doi:10.1371/journal.pntd.0014741)
Supplement: Supplementary file 2 [file pntd.0014741.s002.docx]

| **S2 Table. Parameter estimation for null models at different levels** | | | | | | |
| --- | --- | --- | --- | --- | --- | --- |
| **Type of model** | **Parameter** | **Estimate** | **Standard**  **error** | $\boldsymbol{x}^{\boldsymbol{2}}$ | ***P value*** | **VPCs** |
| One-level model | Fixed part |  |  |  |  |  |
|  | Constant | 0.04 | 0.06 | 0.43 | 0.51 |  |
| Two-level model | Fixed part |  |  |  |  |  |
|  | Constant | 0.04 | 0.10 | 0.14 | 0.71 |  |
|  | Random part |  |  |  |  |  |
|  | Patients level variance | 3.70 | 0.41 | 82.32 | **<0.01** | 0.53 |
|  | Time level variance | 1.00 | 0.00 |  |  |  |
| Three-level model | Fixed part |  |  |  |  |  |
|  | Constant | -0.10 | 0.44 | 0.05 | 0.82 |  |
|  | Random part |  |  |  |  |  |
|  | Hospital level variance | 1.10 | 0.66 | 2.79 | 0.10 | 0.25 |
|  | Patients level variance | 1.14 | 0.22 | 26.38 | **<0.01** | 0.26 |
|  | Time level variance | 1.00 | 0.00 |  |  |  |
