# Supplementary material for: Medication adherence and its determinants among Brucellosis patients in China: A multi-center follow-up study
Source: PLoS Negl Trop Dis. 2026 Oct 5;20(10):e0014741. doi: 10.1371/journal.pntd.0014741 (PMC13637894; doi:10.1371/journal.pntd.0014741)
Supplement: Supplementary file 3 [file pntd.0014741.s003.docx]

| **S3 Table. Distribution of study subjects n (%)** | | | | |
| --- | --- | --- | --- | --- |
| **Region** | **Medical institution** | **Patients**  **N=820** | **Patients at 6 weeks (n=801)** | **Patients at 12 weeks (n=426)** |
| Xinjiang  n=329 | Qapqal Xibe Autonomous County People's Hospital | 137 (16.71%) | 131 (16.35%) | 23 (5.39%) |
|  | Qitai County Centre for Disease Control and Prevention | 192 (23.41%) | 192 (23.97%) | 138 (32.39%) |
| Shandong  n=241 | Shandong Public Health Clinical Center | 151 (18.41%) | 151 (18.85%) | 117 (27.46%) |
|  | Lijin County Yanwo Central Health Centre | 90 (10.98%) | 85 (10.61%) | 63 (14.79%) |
| Inner Mongolia  n=250 | Wengniuqi County Centre for Disease Control and Prevention | 120 (14.63%) | 115 (14.36%) | 45 (10.56%) |
|  | Urad Front Banner County People's Hospital | 130 (15.85%) | 127 (15.86%) | 40 (9.39%) |
