# Supplementary material for: Medication adherence and its determinants among Brucellosis patients in China: A multi-center follow-up study
Source: PLoS Negl Trop Dis. 2026 Oct 5;20(10):e0014741. doi: 10.1371/journal.pntd.0014741 (PMC13637894; doi:10.1371/journal.pntd.0014741)
Supplement: Supplementary file 4 [file pntd.0014741.s004.docx]

**S4 Table. Social support scores across different dimensions**

| **Dimensions** |  | **Patients at 6 weeks**  **(n=801)** | **Patients at 12 weeks**  **(n=426)** |
| --- | --- | --- | --- |
| Objective support score^a^ | $\bar{x}\pm s$ | 9.23 ± 2.38 | 9.34 ± 2.26 |
| Subjective support score^b^ | $\bar{x}\pm s$ | 26.51 ± 3.22 | 26.49 ± 3.14 |
| Support utilization score^c^ | $\bar{x}\pm s$ | 8.06 ± 2.28 | 8.30 ± 2.29 |
| Social support score | $\bar{x}\pm s$ | 43.80 ± 5.79 | 44.12 ± 5.83 |

^a^Objective support, which encompasses the tangible resources and social networks accessible to individuals, including material aid and practical assistance.

^b^Subjective support, which refers to the perceived emotional and psychological backing, such as feelings of being understood, valued, and cared for.

^c^Support utilization, which captures the individual's proactive engagement in seeking and effectively leveraging available support systems, reflecting their capacity to access and utilize resources in times of need.
