# Supplementary material for: Medication adherence and its determinants among Brucellosis patients in China: A multi-center follow-up study
Source: PLoS Negl Trop Dis. 2026 Oct 5;20(10):e0014741. doi: 10.1371/journal.pntd.0014741 (PMC13637894; doi:10.1371/journal.pntd.0014741)
Supplement: Supplementary file 5 [file pntd.0014741.s005.docx]

**S5 Table. The 8-item Morisky Medication Adherence Scale (MMAS-8)**

*Below is a list of statements. Please indicate the extent to which you agree or disagree with them by marking the number of the appropriate box. There are no right or wrong answers. Please give honest answers; otherwise the result would not be valid.*

1. Do you sometimes forget to take your anti-brucellosis medication?

(1) Yes

(2) No

2. Over the past 2 weeks, were there any days when you did not take your anti-brucellosis medication?

(1) Yes

(2) No

3. Have you ever cutback or stopped taking your anti-brucellosis medication without telling your doctor because you felt worse when you took it?

(1) Yes

(2) No

4. When you travel or leave home, do you sometimes forget to bring your anti-brucellosis medication?

(1) Yes

(2) No

5. Did you take your anti-brucellosis medication yesterday?

(1) Yes

(2) No

6. When you feel like your blood pressure are under control, do you sometimes stop taking your anti-brucellosis medication?

(1) Yes

(2) No

7. Do you ever feel hassled about sticking to your treatment plan?

(1) Yes

(2) No

8. How often do you have difficulty remembering to take all your anti-brucellosis medication?

(1) Never/Rarely

(2) Once in a while

(3) Sometimes

(4) Usually

(5) All the time
